# Constitutive Model for Grouted Rock Mass by Macro-Meso Damage

**DOI:** 10.3390/ma16134859

**Published:** 2023-07-06

**Authors:** Yang Liu, Yingchao Wang, Zhibin Zhong, Qingli Li, Yapeng Zuo

**Affiliations:** 1 State Key Laboratory of Intelligent Construction and Healthy Operation & Maintenance of Deep Underground Engineering, China University of Mining Technology, Xuzhou 221116, China; tb19220012b4@cumt.edu.cn (Y.L.); 02210730@cumt.edu.cn (Q.L.); zyp13056251700@163.com (Y.Z.); 2 State Key Laboratory of Geohazard Prevention and Geoenvironment Protection, Chengdu University of Technology, Chengdu 610059, China; 3 School of Mechanics and Civil Engineering, China University of Mining Technology, Xuzhou 221116, China

**Keywords:** constitutive model, grouted rock mass, fractured rock mass, macro-meso damage, composite material

## Abstract

Rock fractures have a significant impact on the stability of geotechnical engineering, and grouting is currently the most commonly used reinforcement method to address this issue. To ensure the stability of grouted rock mass, it is necessary to study its deformation law and mechanical properties. In this study, theoretical analyses and laboratory experiments were conducted, and the fracture width, Weibull model and effective bearing area were introduced to improve the applicability and accuracy of the original damage constitutive model. Moreover, the constitutive model of grouted rock mass was derived by combining it with the mixing law of composite materials. The main conclusions are summarized as follows: (1) Based on macroscopic damage tensor theory, the fracture width parameter was introduced, which effectively described the variation law of macroscopic damage with fracture width to improve the accuracy of the original damage constitutive model. (2) The effective bearing area was used to optimize the original Weibull model to match the stress-strain curve of the rock mass with fractures. (3) The grouting-reinforced rock mass was considered to be a composite material, the original equivalent elastic modulus model was improved by combining macroscopic damage with the Reuss model, and the constitutive damage model of the grouted rock mass was deduced.

## 1. Introduction

In nature, rocks comprise macroscopic geological structural surfaces, such as joints and fractures, and the rock masses cut by them, while macroscopically intact rocks consist of various mineral particles cemented together. Therefore, it is deduced that there are a large number of microscopic defects, such as microcracks and microporous regions, within them. In conclusion, a rock mass is a geological material with compound damage containing both microscopic defects (microscopic damage), such as microcracks and micropores, and macroscopic defects (macroscopic damage), such as joints and fractures. Both of these types of defects affect the mechanical properties of the rock mass based on different mechanisms. Grouting, as one of the common means for reinforcement, has been widely used in various geotechnical projects, such as hydroelectric power stations and underground tunnel construction. During grouting, a slurry flows mainly into the macroscopic fractures to fill the gaps between the bulk of the rock mass, forming a grouted rock mass. In this case, when the rock mass is loaded, the grouted rock is subject to a combination of macroscopic and microscopic damage and grouting reinforcement; therefore, its deformation and damage precisely reflect the coupled cumulative effect of the three factors.

In recent years, there has been considerable research on the mechanical properties of fractured rock masses, with many results achieved. Many experimental studies have been performed by researchers on the stress-strain characteristics of fractured rock masses containing different forms of fractures under load [1,2,3,4,5,6,7,8,9,10,11,12,13,14,15,16,17,18].

For example, Kawamoto et al. [7] explored the stress transfer between compressed fracture surfaces by introducing the nodal pressure transfer coefficient Cv and the shear transfer coefficient Cs. Swoboda et al. [15] investigated the effect of contact between fracture surfaces on stress transfer by introducing the material parameter Hd, reflecting the contact between fracture or fracture surfaces. Xiong and Chen [19] carried out splitting tensile tests on artificially jointed rock mass specimens by using cylindrical samples. These researchers also investigated the effects of the dip angle of the joint plane, radial elastic modulus, radial peak strain, damage variable D, loading rate and high temperature on the tensile strength of the jointed rock mass. Meng et al. [20] proposed a homogenization-based modeling method to study the effects of columnar jointed structures on the mechanical properties of rock masses. A parametric study shows that the elastic parameters of columnar jointed rock masses are strongly dependent on the joint density, thickness, coefficient of variation of the columnar joints and infilled material properties. To investigate the nonlinear energy evolution mechanism, Wang et al. [21] conducted a series of uniaxial compression and acoustic emission (AE) tests on limestone with pores in different shapes. A nonlinear energy self-promotion-inhibition model and a nonlinear energy evolution law for limestone were proposed. The results illustrate the nonlinear energy evolution mechanism of limestone with pores in different shapes. Feng et al. [22] conducted a series of coupled static-dynamic compression experiments on sandstone containing two noncoplanar flaws. The results indicated that the dynamic strength of the flawed sandstone increased first and then decreased at a given dynamic strain rate. Wu et al. [23] employed wire cutting equipment to prepare rock samples with different crossed cracks and then used an acoustic emission system and digital image correlation technique to study the fracture process of rock samples under uniaxial compression. It was found that the strength of rock samples with a single crack is generally larger than that of samples with cross cracks, and the strength changes when the angle of the crack is in a “V” shape.

These experimental and numerical results are expected to improve the understanding of fractured rock masses, which can be used to analyze the stability of rocks and rock structures. At the same time, under the action of ground stress, not only macroscopic fractures but also various microscopic defects are randomly distributed inside the rock, and the minute fractures inside the rock mass expand under the action of an external load. In this case, the mechanical properties of the rock mass gradually deteriorate, which has a great impact on the compressive strength of the rock mass. At present, various internal defects are mainly considered to be random damage. From the perspective of statistical damage mechanics, the degree of damage inside the rock is quantified in terms of microelement strength, and it is assumed that the defects inside the rock obey a certain distribution law before the establishment of the corresponding statistical damage intrinsic model of the rock. Currently, such models mainly consider that the microelement strength of rocks follows different distributions, such as the power function distribution, Weibull distribution or log-normal distribution [2,3,6,7,9,10,11,12,13,14,15,16,17,24,25,26,27,28,29,30,31,32]. Taylor et al. [26] established a computational constitutive model to simulate the dynamic fracture behavior of brittle rock. The essential feature of this model lies in the treatment of the dynamic fracture process in rock as a continuous accrual of damage, and the damage mechanism is based on microcracking in the rock medium. It can be concluded from the results that the model is applicable to the prediction of dynamic rock-fracture behavior. Lee and Ravichandran [33] combined experiments with numerical simulations to explore crack initiation in brittle materials under biaxial static compression with particular attention to the frictional resistance of cracks. In situ photoelastic fringes were obtained and compared with numerical results to determine the stress distribution along crack faces and the identification of slip and stick regions.

The microcracks in rock develop from damage to fracture in a continuous process. To describe this process, Wang et al. [34] proposed a damage-softening statistical constitutive model for rock based on the Weibull distribution of mesoscopic element strength. Current studies on fractured rock masses have been carried out by splitting the two different scales of defects. For a rock mass with macroscopic fractures, only the effects of macroscopic damage are considered. For intact rocks, only the effects of microscopic damage, such as microfractures, are considered, which is clearly not sufficient. The presence of macroscopic fractures during compression may inevitably lead to additional microscopic damage, thereby further deteriorating the mechanical properties of the rock mass. Based on previous research, this paper addresses this problem by coupling the macroscopic damage and the microscopic damage of the rock mass and derives a model that correctly reflects the compression damage of a fractured rock mass.

Most of the current research is focused only on the mechanical properties of the fractured rock itself. In real application scenarios, construction workers usually reinforce fractured rock by grouting. A grouted rock mass, which is formed by the cementation of slurry with rock, is used as the main mechanical carrier during the operation of the project. In engineering, a grouted rock mass is usually stable and will not be destroyed. Therefore, there is little research on fractured rock masses reinforced by grouting. Unfortunately, the damage of a grouted rock mass accounts for most of the disasters that occur during the operation of a project. Therefore, it is necessary to study the mechanical properties of grouted fracture masses. A slurry flows along and fills the macroscopic fractures and combines with the rock mass to form a grouted rock mass as a standard composite material. For the equivalent elastic modulus of composites, the Voigt model and Reuss model are most commonly used. However, the equivalent modulus of elasticity for composites is mainly applied to metals and composite materials and has rarely been used in geotechnical engineering [35,36,37,38,39,40,41,42,43].

This paper explores the mechanical properties of grouted rock masses. Based on the idea of coupling macro-meso damage, the macroscopic damage tensor and microscopic damage random distribution model are combined, and influencing factors such as fracture width and effective bearing area are introduced to improve the applicability and accuracy of the original damage constitutive model. Moreover, the damage constitutive model of a grouted rock mass is established by using the composite material mixing law. The new model can accurately describe the deformation and damage characteristics of the fractured rock mass reinforced by grouting.

## 2. Establishment of a Constitutive Model for Grouted Rock Mass

### 2.1. Damage Mechanism of the Fractured Rock Mass

Rock masses under high ground stress often have a large amount of internally distributed microscopic damage, such as microfractures and pores, and macroscopic damage, such as large fractures and joints. The coupling effect of these two types of damage greatly deteriorates the mechanical properties of fractured rock masses, from which the construction of deep underground projects may suffer. The stability of an engineering structure built in or on rock masses is under the direct influence of the discontinuous factors of the rock masses. For discontinuous factors, the concept of a damaged rock mass has been introduced, and the damage mechanics theory for the mechanics of discontinuous rock masses has been proposed.

The following assumptions are made for both macroscopic and microscopic fractures in the rock mass.

(1) The undamaged part of the rock mass during the test is always a linear elastic material.
(2) Macroscopic fractures can be directly observed by using the naked eye, which can be the main feature used to distinguish macroscopic fractures and microscopic fractures. In addition, based on the distribution characteristics of macroscopic and microscopic fractures, macroscopic fractures are equated to anisotropic damage, while microscopic fractures are equated to isotropic damage in the study.
(3) The microscopic damage and macroscopic damage are described by different methods, respectively. The microscopic damage can be defined as the damage caused by loading. The macroscopic damage is defined as the initial damage caused by the precast fractures to the rock, and the initial macroscopic damage does not change during the loading process.

**Figure 1:**
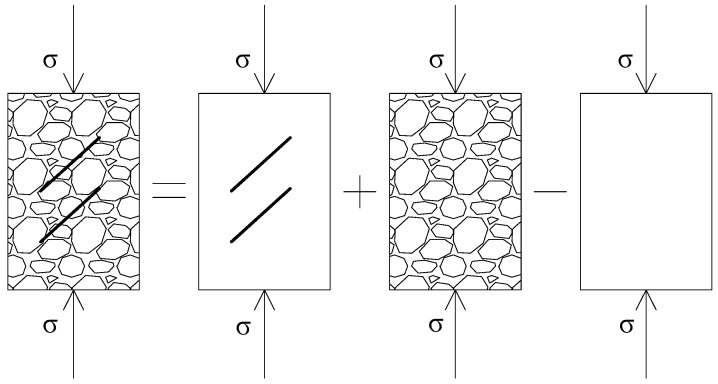
Schematic diagram of calculated equivalence strain.

Macro-meso coupled damage is not the simple addition of microscopic damage and macroscopic damage; macroscopic damage and microscopic damage will influence each other during the loading process and cause some additional damage. Therefore, the study of macro-meso coupling damage should be based on the Lemaitre assumption, that is, the strain equivalence principle. The Lemaitre assumption was proposed by French scholar Roger Lemaitre in 1982. Since then, the Lemaitre assumption has been widely used in the fields of geotechnical engineering and material mechanics [44,45,46]. Figure 1 illustrates a rock containing both macroscopic and microscopic defects, a rock containing only macroscopic defects, a rock containing only microscopic defects and a virtual rock without damage at all. Their moduli of elasticity are $E_{12}$, $E_{1}$, $E_{2}$ and $E_{0}$, respectively, and their strains under stress σ are $\varepsilon_{12}$, $\varepsilon_{1}$, $\varepsilon_{2}$ and $\varepsilon_{0}$, respectively. Coupled damage theory can be described by the following equations:

(1) $$\varepsilon_{12}=\varepsilon_{1}+\varepsilon_{2}-\varepsilon_{0}$$

Therefore, Equation (1) can be changed to the following:

(2) $$\frac{\sigma }{E_{12}}=\frac{\sigma }{E_{1}}+\frac{\sigma }{E_{2}}-\frac{\sigma }{E_{0}}$$

The microscopic damage during rock mass loading is defined as damage $D_{1}$, the macroscopic damage caused by the prefabricated fracture is defined as damage $D_{2}$ and the coupled damage during the loading of the fractured rock mass is defined as $D_{12}$.

Based on the Lemaitre assumption:

(3) $$\{\begin{array}{c} E_{12}=E_{0}\left(1-D_{12}\right)\\ E_{1}=E_{0}\left(1-D_{1}\right)\\ E_{2}=E_{0}\left(1-D_{2}\right) \end{array}$$

Substituting Equation (3) into Equation (2), the following can be obtained:

(4) $$D_{12}=1-\frac{\left(1-D_{1}\right)\left(1-D_{2}\right)}{1-D_{1}D_{2}}$$

As shown in Equation (4), when the rock contains only microscopic damage, i.e., $D_{12}=D_{1}$, the coupled damage at this time is equal to the microscopic damage. When the rock contains only macroscopic damage, i.e., $D_{12}=D_{2}$, the coupled damage at this time is equal to the macroscopic damage, which is consistent with the actual situation. Therefore, the macro-meso coupling damage established by using this method is reasonable.

### 2.2. Damage Constitutive Model of Grouted Rock Mass

#### 2.2.1. Microscopic Damage Model of the Fractured Rock Mass

The rock masses are internally distributed with a large number of microscopic fractures, which can be considered to be random damage. Based on the idea of statistical damage mechanics, the internal microscopic fractures of rock are quantified by microelement strength. According to the distribution characteristics of microscopic damage within the rock mass, it is assumed that the microscopic damage conforms to a certain statistical model, and then the corresponding statistical damage constitutive model can be established.

Assuming that the number of damaged microelements under certain strain ε is s, the ratio of the number of damaged microelements to the total number of microelements S is defined as the microscopic damage $D_{1}$.

(5) $$D_{1}=\frac{s}{S}\;$$

Under a certain strain ε, the number of damaged microelements s is:

(6) s=S·P(ε)

where P(ε) is the statistical model of damaged microelements.

Duarte et al. [47] found that the Weibull distribution is the appropriate statistical model of the grain size and grain shape. Since then, the Weibull statistical model has been used to characterize the heterogeneity of different materials based on the concept that microscopic defects within a material determine its mechanical strength [31,48,49].

It is assumed that the damaged microelement obeys the Weibull distribution and that its probability density function is:

(7) $$P\left(\varepsilon \right)=\frac{m}{\varepsilon_{0}}{\left(\frac{\varepsilon }{\varepsilon_{0}}\right)}^{m-1}e^{-{\left(\frac{\varepsilon }{\varepsilon_{0}}\right)}^{m}}$$

where P(ε) is the statistical model of damaged microelements, and $m\;\mathrm{and}\;\varepsilon_{0}$ are distribution parameters.

In combination with Equations (6) and (7), the number of damaged microelements s is:

(8) $$s=\int_{0}^{\varepsilon }SP\left(\varepsilon \right)d\varepsilon =S\left(1-e^{-{\left(\frac{\varepsilon }{\varepsilon_{0}}\right)}^{m}}\right)$$

The microscopic damage evolution equation of the loaded rock with strain can be obtained as:

(9) $$D_{1}=\frac{s}{S}=\int_{0}^{\varepsilon }P\left(\varepsilon \right)d\varepsilon =1-e^{-{\left(\frac{\varepsilon }{\varepsilon_{0}}\right)}^{m}}$$

**Figure 2:**
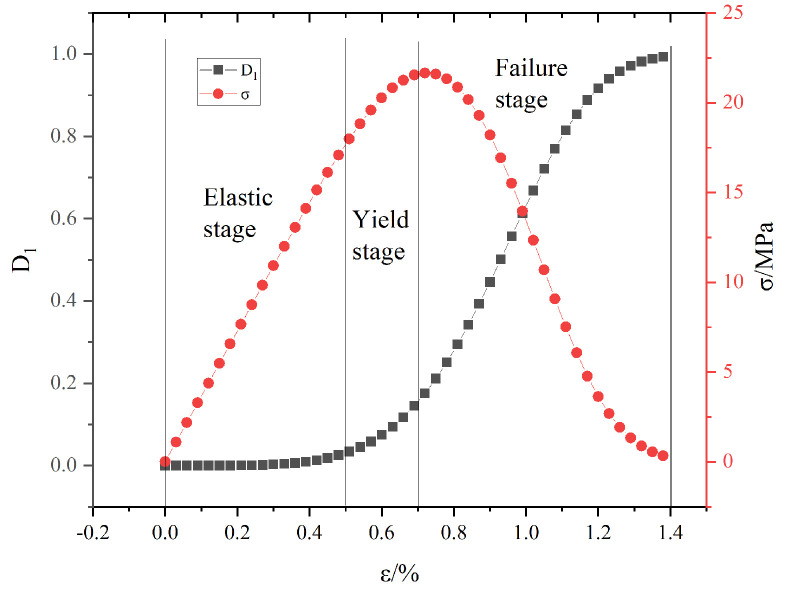
Weibull model schematic.

Figure 2 shows the evolution law of microscopic damage $D_{1}$ and the stress-strain curve at $\varepsilon_{0}$ = 1.3, m = 1.65 and E = 3650. As seen from the figure, the Weibull model can effectively describe the elastic stage and yield stage of the stress-strain curve. However, due to the limitations of the Weibull model itself, its cumulative probability distribution function is always monotonically increasing between (0, +∞), which makes it unable to accurately describe the compaction at early stage of loading. This was also found in a previous research [50].

The deformation of rock under load can be divided into two types: one is caused by the deformation of the rock mass, and the other is caused by the compression of pores. When the stress is small, the two kinds of deformation occur simultaneously, and the rock is in the compaction stage. With increasing stress, the internal pores of the rock are completely closed. At this time, the deformation is only caused by the rock, and the rock enters the elastic stage.

During the loading process, the apparent area of the rock mass cross section is A. Due to the existence of initial pores, the pore area of the cross section is $A_{2}$. Therefore, the effective bearing area $A_{1}=A$ − $A_{2}$, and then the effective stress $\sigma^{*}$ is:

(10) $$\sigma^{*}=\frac{P}{A_{1}}\;\sigma =\frac{P}{A_{1}+A_{2}}=\sigma^{*}\cdot \frac{A_{1}}{A_{1}+A_{2}\;}$$

where P is the total pressure on the rock mass.

The ratio of the effective bearing area to the apparent area of the rock mass cross section is assumed to be:

(11) $$K=\frac{A_{1}}{A_{1}+A_{2}\;}$$

Then:

(12) $$\sigma =\sigma^{*}\cdot K=\varepsilon \cdot \left(1-D_{1}\right)\cdot E\cdot K\;$$

When the stress is small, $K=\frac{A_{1}}{A_{1}+A_{2}\;}$, and with increasing stress, the internal pores of the rock mass gradually close and $A_{2}$ continuously decreases. At the end of the compaction stage, K = 1. Therefore, the following assumption can be made:

(13) $$K=\frac{1}{1+exp\left(c\varepsilon +d\right)}$$

where ε is the strain and c and d are distribution parameters.

**Figure 3:**
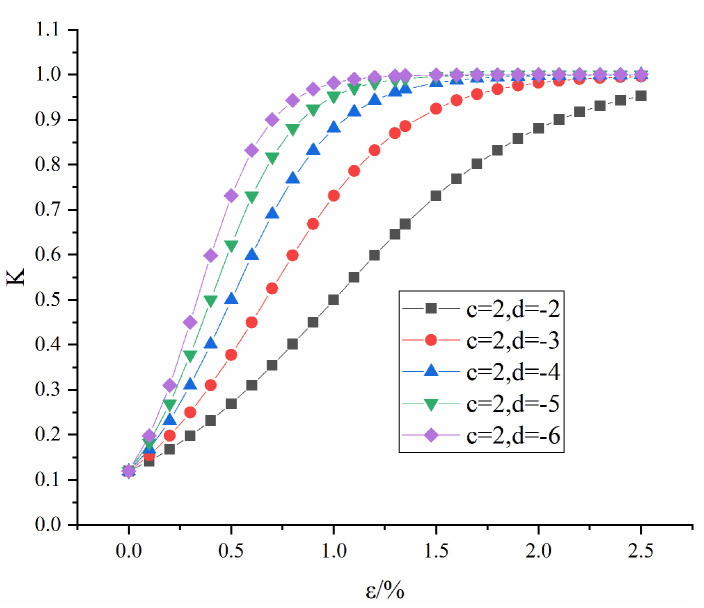
Influence of parameter d on K.

Figure 3 shows the influence of d on the evolution law of K when c = 2. As shown in Figure 3, the value of K starts from 0.1, increases with strain and eventually tends to 1, indicating that K conforms to the evolution law of rock mass pores during the compaction process. With increasing d, the initial value of K remains unchanged, but the curve becomes steeper, which indicates that the increase in d makes the duration of the compaction stage of the rock mass increase and that the rock transitions from brittleness to ductility.

**Figure 4:**
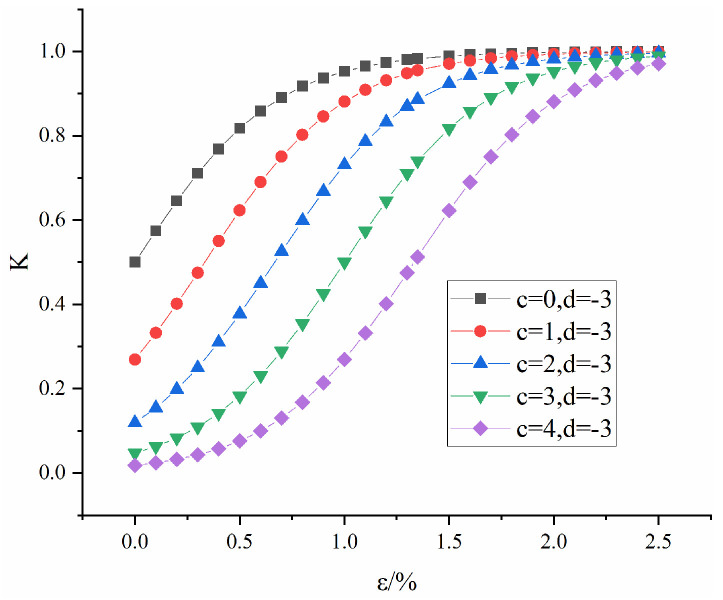
Influence of parameter c on K.

Figure 4 shows the influence of c on the evolution law of K when d = −3. As shown in Figure 4, with increasing c, the growth rate of the curve remains unchanged, but the initial value of K decreases, indicating that the increase in c increases the variation range of the compaction stage. It is believed that c reflects the concentration of the pore distribution inside the rock mass.

**Figure 5:**
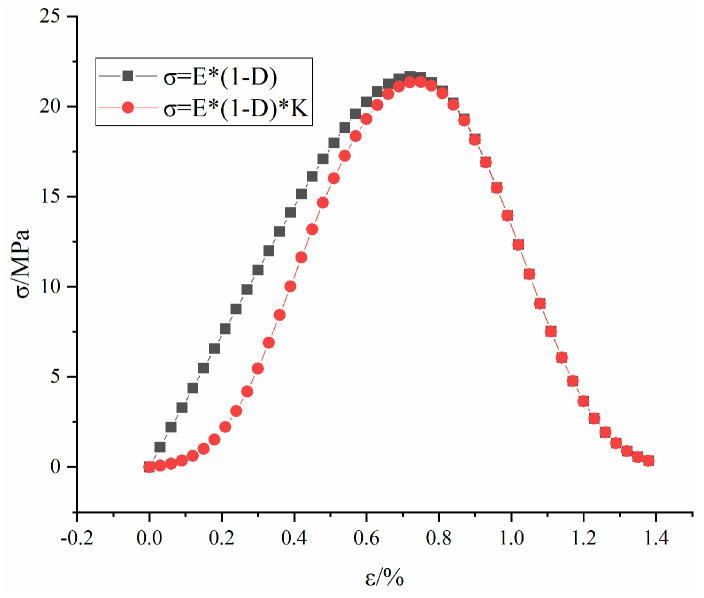
Stress−strain curve of rock mass with or without K.

Figure 5 shows the stress-strain curve with or without K at $\varepsilon_{0}$ = 1.3, m = 1.65, E = 3650, c = −10 and d = 3. As seen from the figure, the improved Weibull model can accurately describe the process of transition from the compression stage to the elastic stage, yield stage and damage stage during the compression process of the rock mass. It overcomes the shortcomings of the original Weibull model and is closer to reality.

#### 2.2.2. Macroscopic Damage Model of a Fractured Rock Mass

According to damage theory, the definition of damage is both the premise and basis of damage model establishment. Some scholars [14,51] believe that the fractures in rock masses generally have a certain directionality in spatial distribution in most cases. The macroscopic damage of fractured rock masses can be defined according to the geometric parameters of fractures, such as distribution spacing and geometric size.

**Figure 6:**
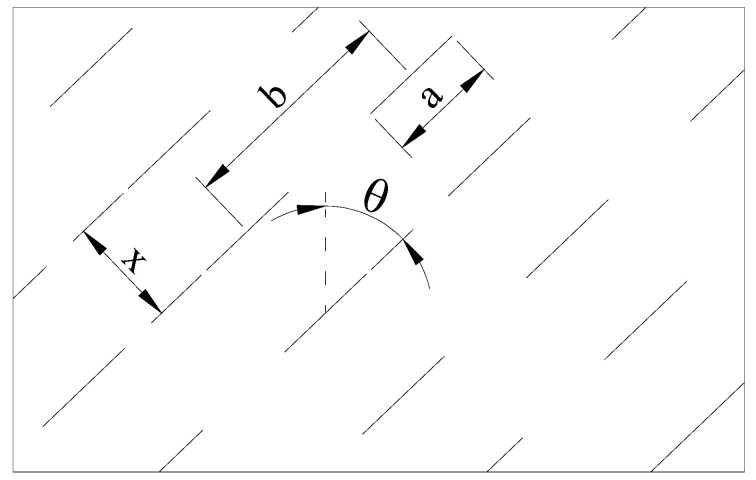
Geometric parameters for fractures [14].

The spatial geometric parameters of fractures are shown in Figure 6. These parameters include the fracture area a, the fracture layer distance x, the fracture center distance b and the fracture dip angle θ. These fundamental geometric quantities determine the degree of fracture influence on the mechanical properties of the rock mass. Therefore, the damage of the rock mass Ω should be a function of these basic geometric parameters, calculated as [14]:

(14) $$\Omega =\left[1-exp\left(-\frac{a}{bx}\right)\right]$$

**Figure 7:**
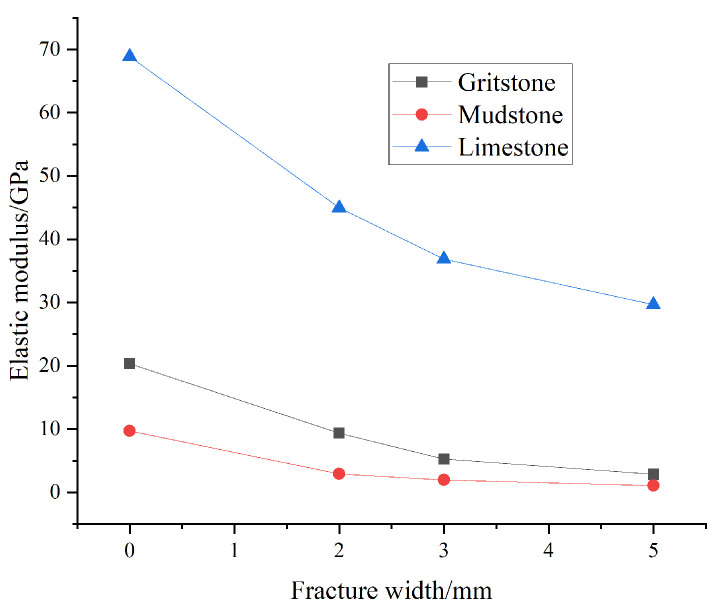
Curve of elastic modulus with fracture width [53].

The macroscopic damage model described above can effectively describe the weakening effect of fractures on the elastic modulus of rocks. However, in previous studies, the width of the fracture is usually assumed to be zero, thereby ignoring the effect caused by the width of the fracture. It is known from the previous studies [52,53] that the fracture width has a significant impact on the mechanical properties of the rock. In particular, the injection of slurry will inevitably lead to the deformation and opening of the original fractures. In view of this, the fracture width should not be neglected in the study of the damage constitutive model. According to the reference, the effect of the fracture width on the mechanical properties of the rock mass is shown in Table 1 and Figure 7, and the equivalent elastic modulus of the fractured rock mass decreases nonlinearly with increasing fracture width.

Based on Equation (14), the fracture width r is introduced to modify the macroscopic damage model. Assume that the macroscopic damage considering fracture width is f(Ω). According to the damage law, when the fracture width r tends to 0, f(Ω) tends to Ω. Similarly, when the fracture width r tends to infinity, f(Ω) tends to 1. Finally, the modified macroscopic damage model f(Ω) is presented as follows:

(15) f(Ω)=1−(1−Ω)·exp(pr)

where p represents the coefficient that is less than 0.

#### 2.2.3. Effect of Grouting Reinforcement

To improve the mechanical properties of fractured rock mass, grouting reinforcement technology is often applied. The purpose is to let the cement slurry with good cementing properties and certain load-bearing capacity fill the fractures of the rock to form a grouted rock mass. The grouted rock mass is a composite material, which conforms to the “mixing law” of the elastic modulus of the composite materials. Macroscopic defects, microscopic defects and solidified slurry in the fractured rock mass after grouting reinforcement jointly affect the mechanical properties of the grouted rock mass. Therefore, further research on the coupled damage of grouted rock masses is necessary.

Since the slurry mainly fills in the macroscopic fractures during grouting and cements the rock mass to form a grouted rock mass, the reinforcement effect of grouting should be related to macroscopic damage. The equivalent elastic modulus of the grouted rock mass is derived by combining the macroscopic damage theory with the “mixing law” of the equivalent elastic modulus.

In general, the mechanical properties of composites depend on the content, bonding force, shape and spatial distribution of each phase. The above macroscopic damage model expresses the maximum macroscopic damage in the normal direction of fracture, and each phase bears equal stress in the composite material. The Reuss model is a common material constitutive model used to describe the mechanical behavior of heterogeneous materials. This model was proposed by G. Reuss in 1929 and has been widely used in the field of material mechanics. Therefore, based on the Reuss model, the equivalent elastic modulus formula of the grouting rock mass can be obtained by replacing each phase with the macroscopic damage model [35,36]:

(16) $$E={\left(\frac{f\left(\Omega \right)}{E_{a}}+\frac{1-f\left(\Omega \right)}{E_{2}}\right)}^{-1}$$

where E refers to the equivalent elastic modulus of the composite material and $E_{a}$ stands for the elastic modulus of the grouting material.

Then, the macroscopic damage model of the grouted rock mass is described as follows:

(17) $$F\left(\Omega \right)=1-\frac{E}{E_{0}}=1-\frac{{\left(\frac{f\left(\Omega \right)}{E_{a}}+\frac{1-f\left(\Omega \right)}{E_{2}}\right)}^{-1}}{E_{0}}$$

The macroscopic fractures in the rock mass lead to obvious anisotropic characteristics, and the macroscopic damage F(Ω) described above is only the damage in the normal direction of fracture. To describe the damage of fractured rock masses in different directions, the macroscopic damage F(Ω) must be tensorized to make the original macroscopic damage more widely applicable. Therefore, the damage tensor N is introduced. Then, the final macroscopic damage model of the grouted rock mass is described as follows. To describe the damage of fractured rock masses in different directions, the method proposed by Kawamoto et al. [7] is used to introduce the damage tensor N. N is a second-order symmetric tensor, which is calculated by:

(18) $$N=n⊗n=\left[\begin{array}{cc} sin^{2}\theta & sin\theta cos\theta \\ cos\theta sin\theta & cos^{2}\theta \end{array}\right]$$

where θ represents the angle between the fracture and the *Y*-axis, and n represents the unit normal vector of the fracture.

(19) $$D_{2}=F\left(\Omega \right)\cdot N=F\left(\Omega \right)\cdot \left[\begin{array}{cc} sin^{2}\theta & sin\theta cos\theta \\ cos\theta sin\theta & cos^{2}\theta \end{array}\right]$$

According to the damage theory, when the rock mass contains both macro- and microscopic damage, the changes in coupling-damage variables are reflected in the damage model. Therefore, by replacing the microscopic damage $D_{1}$ in Equation (4) with Equation (9), the macroscopic damage $D_{2}$ in Equation (4) is replaced with Equation (19). The constitutive model for grouted rock mass by macro-meso damage can be obtained as follows:

(20) $$\sigma =E_{0}\frac{\left(1-D_{1}\right)\left(1-D_{2}\right)}{1-D_{1}D_{2}}K\varepsilon$$

where σ, ε and $E_{0}$ are, respectively, the stress, strain and elastic modulus in the form of tensors.

## 3. Experiment and Model Validation

### 3.1. Test Equipment and Sample Preparation

To verify the validity of the constitutive model established above, uniaxial compression tests were carried out on intact rock and grouted rock mass samples. The test results were analyzed and compared with the calculated results to investigate the stress-strain curves, compressive strength and peak strain of grouted rock mass containing prefabricated fractures of different forms.

The loading was carried out under the displacement control mode at a loading speed of 0.001 mm/s. The test instrument used is a CMT5205/5305 Material Testing Machine, which was purchased from Metus Industrial Systems Co., Ltd., Shenzhen, China, and the load cell capacity of the machine 0–300 KN.

**Figure 8:**
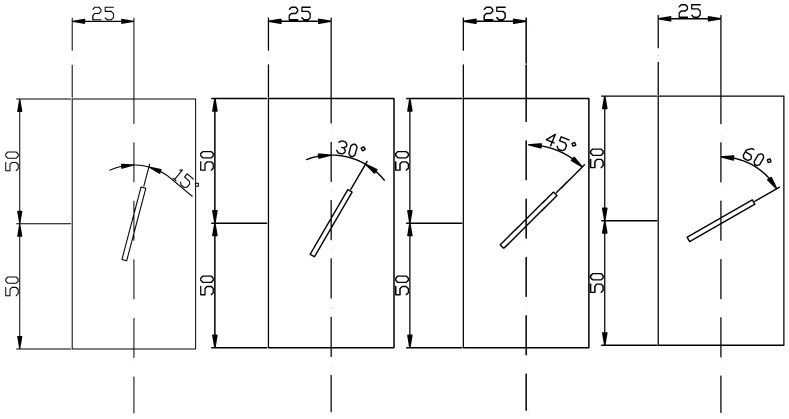
Schematic diagram of fractured rock mass.

(1) A cubic specimen of intact red sandstone with a standard size, i.e., width × height of 50 mm × 100 mm, was produced. The cubic specimens were provided by Roke New Material Technology Co., Ltd., Chongqing, China, which independently developed automatic rock sample coring equipment and cooperates with many universities and scientific research institutions.
(2) The test specimens were divided into intact specimens and prefabricated fracture specimens. Non-through fractures were cut in the complete specimens by using a waterjet at fracture angles of 15°, 30°, 45° and 60°; a width of 2 mm; and a length of 30 mm, as shown in Figure 8.
(3) PO42.5 concrete was used as the grouting material, which was purchased from China Zhucheng Yangchun Cement Co., Ltd., Weifang, China, whose products passed the ISO9001 quality management system certification. The concrete was injected into the precast fractures and allowed to stand for 48 h, and then the specimens were subjected to standard curing for 28 d before being removed for use. The experimental test was repeated 3 times for each kind of sample, and the average value was calculated as the test result.

### 3.2. Test Results

#### 3.2.1. Rock Mass Crack Propagation

**Figure 9:**
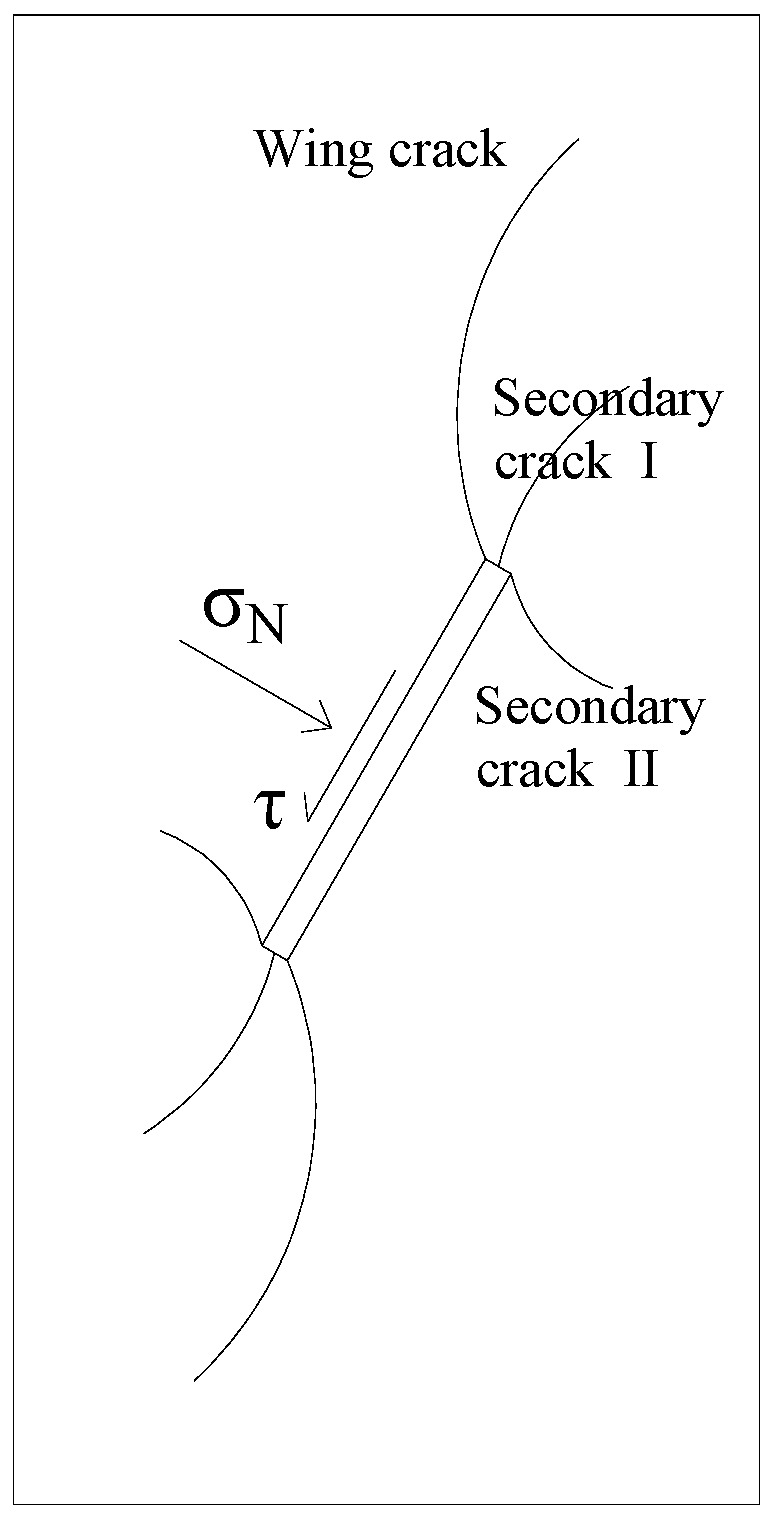
Simplified model of single fractured rock.

**Figure 10:**
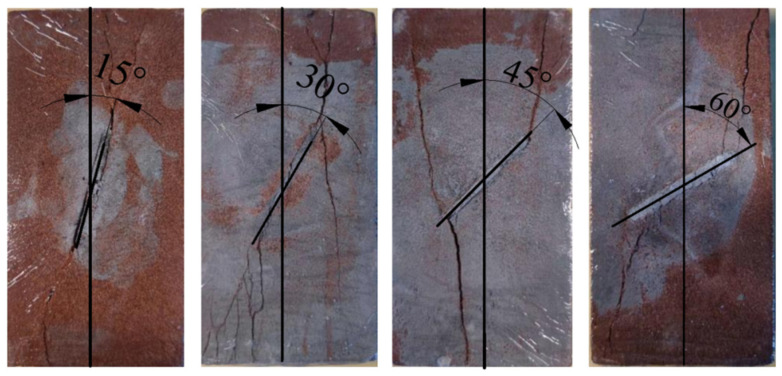
Schematic diagram of rock mass cracking.

The newly emerged cracks at or adjacent to the fracture tip are classified into winged cracks and secondary cracks. The stress state model of the rock containing prefabricated fractures in uniaxial compression is shown in Figure 9. According to linear elastic fracture mechanics, when the stress at the fracture tip is less than the critical stress, no crack extension occurs. When the stress at the tip is greater than or equal to the critical stress, wing cracks are produced at the fracture tip and occur at the two initial fracture ends. The wing crack is the initial crack, which appears first and then continues to develop in the form of a curve along the direction of the maximum principal stress as the load increases. The crack has a narrow and smooth surface with a moderate development trend, which is a tension crack. Secondary cracks are also produced at or near the tip of the prefabricated fracture, which are divided into Types I and II according to the direction of the crack. The secondary cracks show a zigzag crack trend and significant signs of friction on the crack surface and rough surface, and the shear stress has a driving effect on the expansion of the secondary cracks, which can be characterized as shear damage, as shown in Figure 10.

As shown in Figure 10, when the dip angle of the prefabricated fracture is 15° and 60°, the crack is a wing crack, indicating that the specimen damage is mainly tension damage at this time. When the prefabricated fracture is 30° and 45°, not only wing cracks but also obvious Type II secondary cracks appear. This is because the friction angle of the rock is between 30° and 45°, and the specimen damage at this time is closer to compression-shear damage, leading to the generation of secondary fractures.

#### 3.2.2. Test Results Analysis

**Figure 11:**
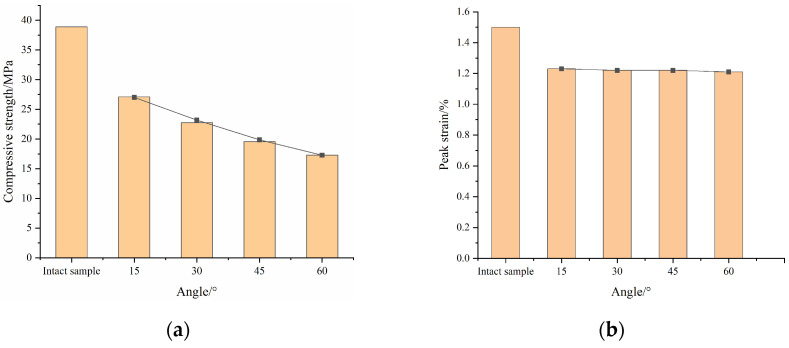
Test results: (**a**) compressive strength; (**b**) peak strain.

Table 2 and Figure 11 show the specimens’ compressive strength and peak strain. It can be seen from the figures that the presence of fractures causes a decrease in compressive strength and peak strain of the grouted rock mass compared with the intact samples. The compressive strength of the intact sample was 38.9 MPa, while that of the grouted rock mass was 27.1 MPa at 15°, 22.76 MPa at 30°, 19.57 MPa at 45° and 17.26 MPa at 60°. The peak strain was 1.52% for the intact sample, 1.23% at 15°, 1.22% at 30°, 1.21% at 45° and 1.21% at 60°. When macroscopic damage is present in the rock mass, both the strength and stiffness are weakened.

### 3.3. Validation of the Constitutive Model

**Figure 12:**
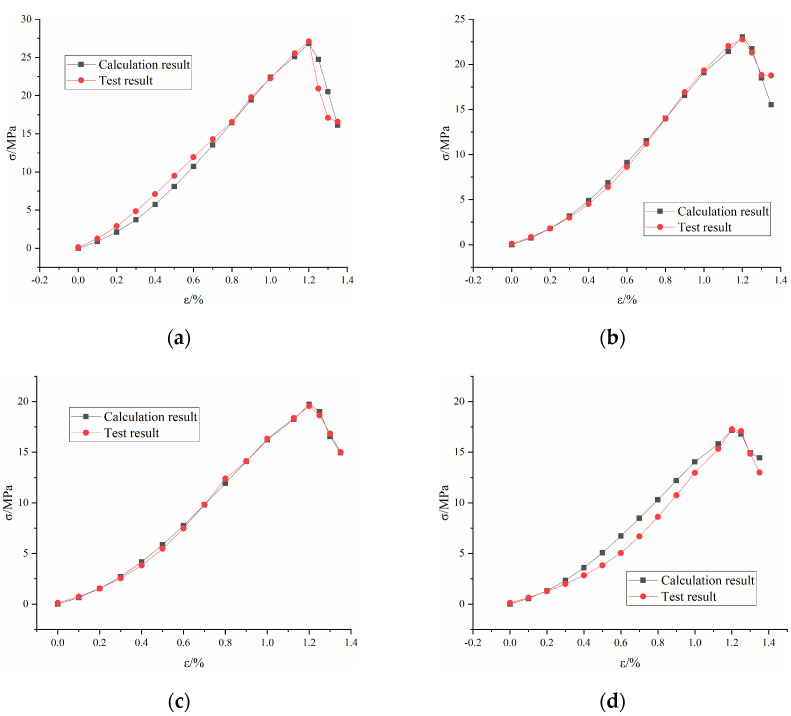
Comparison between stress−strain calculation curve and test curves: (**a**) 15°, (**b**) 30°, (**c**) 45° and (**d**) 60°.

The stress-strain calculation curve and test curve of the grouted rock mass are shown in Figure 12. The detailed calculation parameters are shown in Table 3. Elastic modulus of rock ($E_{0}$) can be obtained by uniaxial compression of the intact rock sample. Elastic modulus of grouting material ($E_{a}$) can be obtained by a uniaxial compression test of a self-made concrete sample. The unknown mechanical parameters m, $\varepsilon_{0}$, c and d of the intact rock are calculated by the inversion of Equations (9) and (13). The unknown mechanical parameters p of the fractured rock mass are calculated by the inversion of Equations (15), (17) and (19).

**Figure 13:**
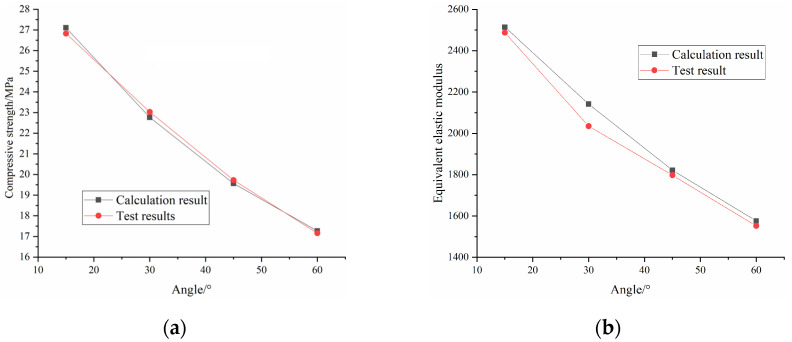
Comparison between calculation results and test results: (**a**) compressive strength; (**b**) equivalent elastic modulus.

Figure 13 shows the comparison of the compressive strength and equivalent elastic modulus between the test results and calculated results. It can be seen from the comparison that the constitutive model established in this paper can well simulate the stress-strain curve of the grouted rock mass and reproduce the brittle characteristics of red sandstone. The average error percentage of compressive strength is 2.19%. The average error percentage of the equivalent elastic modulus is 2.32%. This indicates that the new damage model can reflect the strength and deformation characteristics of grouted rock mass.

### 3.4. Validation of the Constitutive Model

#### 3.4.1. Evolution of Microscopic Damage

**Figure 14:**
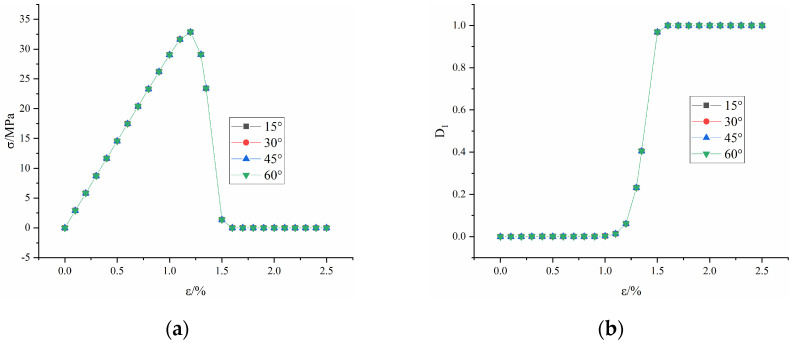
Curve of compressive strength and $D_{1}$ with strain under different fracture dip angles without considering macroscopic damage: (**a**) compressive strength; (**b**) $D_{1}$.

Figure 14 shows the evolution law of compressive strength and microscopic damage $D_{1}$ with strain without considering macroscopic damage, respectively. The calculation results are consistent with the variation pattern of the actual stress-strain curve, indicating that the microscopic damage model used in this paper is reasonable and effective.

It can also be seen from Figure 14 that although the microscopic damage model used in this paper can simulate the stress-strain curve, the microscopic damage curves with different fracture dip angles coincide together. This finding indicates that the microscopic damage model cannot reflect the influence of prefabricated fractures on the rock mass.

#### 3.4.2. Evolution of Macroscopic Damage

**Figure 15:**
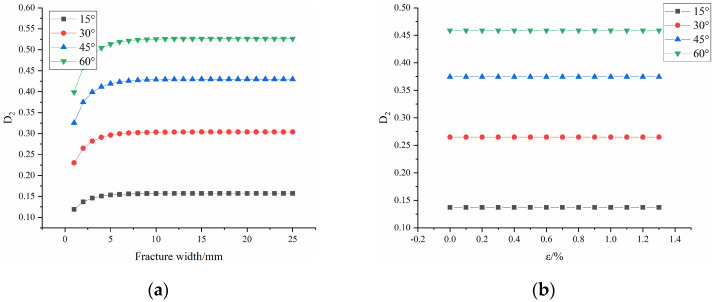
Curve of $D_{2}$ under different fracture dip angles: (**a**) with fracture width; (**b**) with strain.

**Figure 16:**
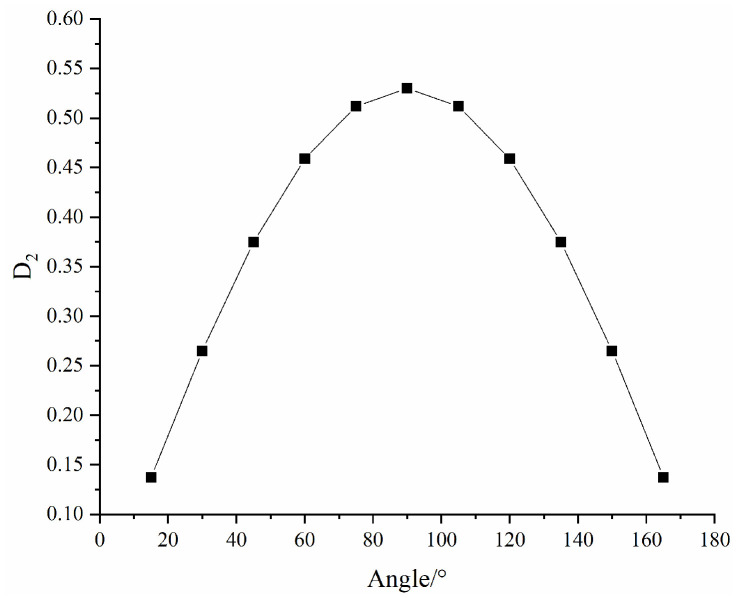
Curve of $D_{2}$ with fracture dip angle.

Figure 15 shows the evolution law of macroscopic damage $D_{2}$ with fracture width and with strain under different fracture dip angles. Figure 16 shows the evolution law of macroscopic damage $D_{2}$ with fracture dip angles.

As shown in Figure 15a, the macroscopic damage $D_{2}$ increases first before stabilizing with fracture width. Due to the tensorization of the macroscopic damage, the damage value eventually stabilizes at 0.15731 for 15°, 0.3039 for 30°, 0.42979 for 45° and 0.51638 for 60°. This is consistent with the reference [14], that is, the fractures have a greater impact on the compressive strength of the rock when they first appear, and this influence gradually decreases with increasing fracture width. Eventually, the macroscopic damage caused by the fractures to the rock mass will stabilize at a specific value.

As shown in Figure 15b, the macroscopic damage $D_{2}$ does not change with increasing strain. This is consistent with assumption 3 described previously, where macroscopic damage $D_{2}$ is defined as the initial damage caused by the precast fractures to the rock and the initial macroscopic damage does not change during the loading process. 

As shown in Figure 16, with increasing fracture dip angle, the macroscopic damage first increases and then gradually decreases, peaking at 90°. The curve is similar to the sinθ function curve. Correspondingly, the pattern of change in the equivalent modulus of elasticity and compressive strength of the rock mass obtained from the tests is also in agreement with that in the macroscopic damage values, indicating that the relationship between the macroscopic damage model and fracture width and fracture dip angle established in this paper is reasonable and valid.

**Figure 17:**
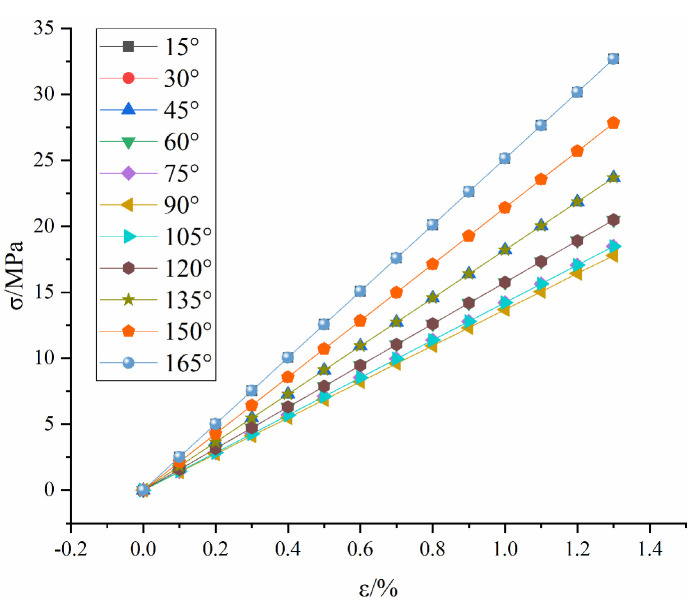
Stress−strain curve without considering microscopic damage.

**Figure 18:**
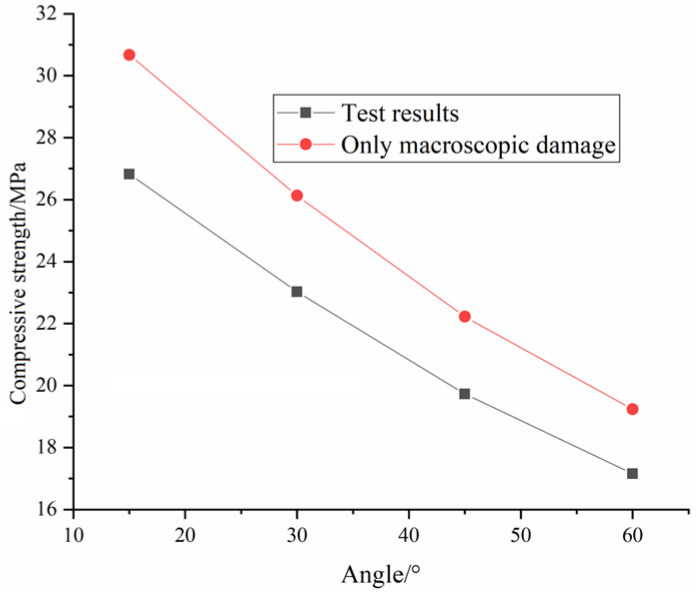
Comparison between the calculated results of compressive strength considering only $D_{2}$ and the test results.

Figure 17 shows the stress-strain curve when only macroscopic damage $D_{2}$ is considered. The stress-strain curve is a straight line, which cannot describe the changes in stress-strain at each stage. Figure 18 shows the comparison between the calculated results of compressive strength considering only macroscopic damage and the test results. It can be clearly seen that the calculation results can reflect the influence of the fracture angle on the compressive strength but the calculated results are obviously larger than the test results. This indicates that it is not enough to consider the macroscopic damage alone.

#### 3.4.3. Evolution of Coupled Damage

**Figure 19:**
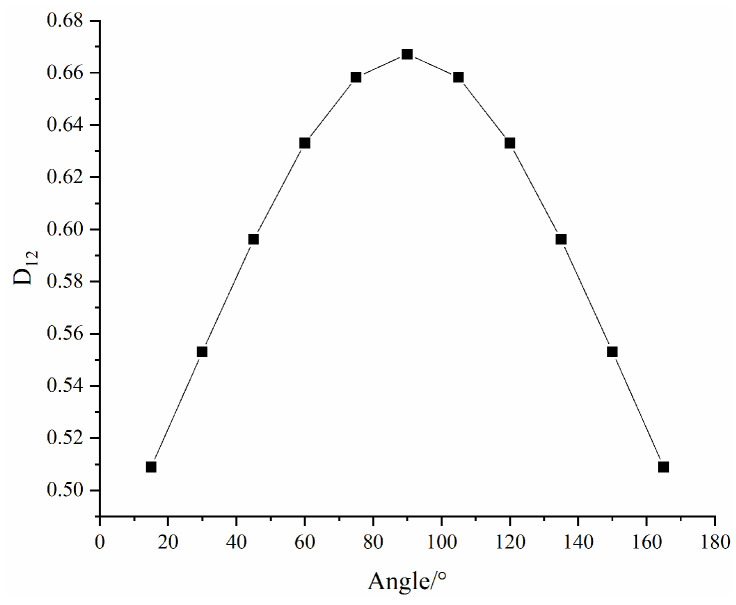
Curve of $D_{12}$ with fracture dip angle.

Figure 19 shows the evolution law of coupling damage $D_{12}$ with fracture angle. For ε = 1.4%, the coupling damage $D_{12}$ rises and then falls with the fracture angle.

**Figure 20:**
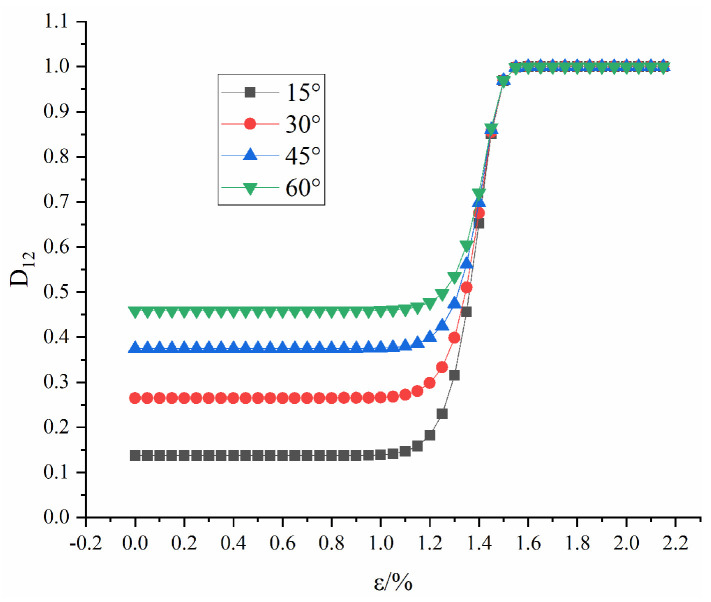
Curve of $D_{12}$ with strain under different fracture dip angles.

Figure 20 shows the evolution law of coupling damage $D_{12}$ with strain. As shown in Figure 20, before the strain is 1.0%, $D_{12}$ is basically unchanged and the coupling damage $D_{12}$ is equal to the macroscopic damage $D_{2}$. After the strain is 1.0%, $D_{12}$ gradually rises with increasing strain, and the curve becomes steeper as the angle decreases and finally converges to 1. The coupled damage takes into account the effects of macroscopic damage and microscopic damage on the stress-strain curve, and the coupling of the two causes a certain degree of additional damage. Combining Figure 14, Figure 18 and Figure 20, it can be seen that macroscopic damage mainly affects the compressive strength of the rock mass and that microscopic damage mainly affects the curve changes at different stages of the rock mass, neither of which alone can accurately describe the stress-strain curve. 

#### 3.4.4. Evolution of the Grouting Reinforcement Effect

**Figure 21:**
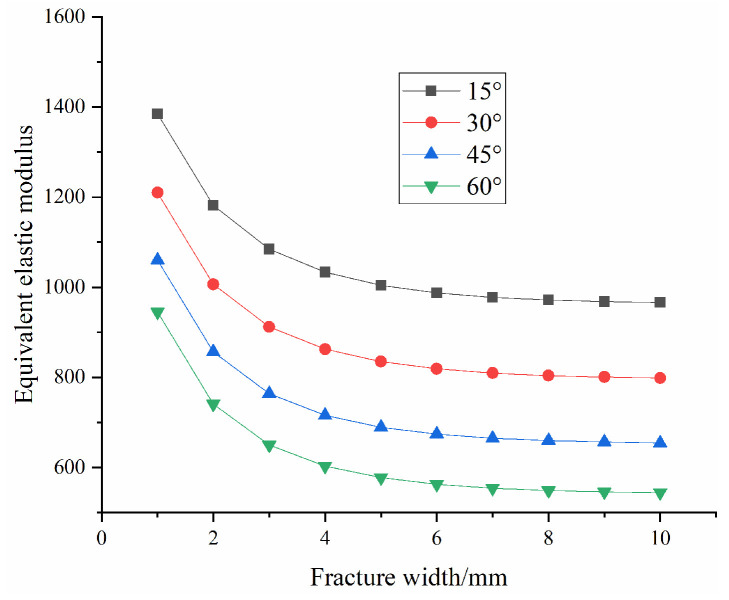
Curve of equivalent elastic modulus with fracture width under different fracture dip angles.

Based on the calculation of the damage constitutive model established in this paper, Figure 21 shows the evolution law of the equivalent elastic modulus with fracture width. The comparison between Figure 21 and Figure 7 indicates that the calculation results of the coupled damage model established in this paper are in agreement with those of the reference [53] and that the equivalent elastic modulus decreases exponentially with increasing fracture width, showing the validity of the model.

**Figure 22:**
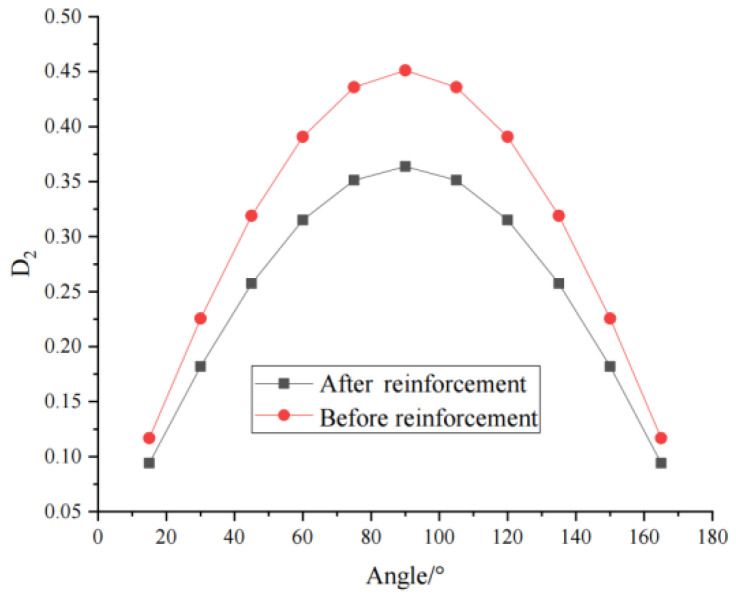
Curve of $D_{2}$ before and after reinforcement.

Figure 22 shows the evolution law of the macroscopic damage curve before and after grouting reinforcement based on the model derived in this paper. It can be seen from the figure that the macroscopic damage is significantly reduced after grouting reinforcement. The larger the fracture dip angle is, the better the grouting reinforcement effect, and the greater the effect of grouting reinforcement on macroscopic damage. The reason is that the effective contact area occupied by the grouting material becomes larger with decreasing fracture dip angle, so the grouting reinforcement effect is better. The model established in this paper accurately reflects the repair effect of grouting reinforcement on fractured rock masses.

## 4. Conclusions

In this paper, based on previous research, the fracture width and effective bearing area are considered in the damage constitutive model for the first time, and the Weibull model is used to describe the evolution law of microscopic damage with load. Moreover, the existing composite elastic modulus mixing law and macroscopic damage model are combined to deduce the constitutive model for grouting rock masses. The main conclusions are drawn as follows:

(1) Based on the fact that the rock mass contains both macroscopic and microscopic damage, the original coupled damage model was improved by introducing fracture width, effective bearing area and Weibull model and by combining damage mechanics and fracture mechanics. The grouted rock mass was considered to be a composite material. The damage constitutive model of the grouting rock mass was derived by combining the elastic modulus mixing law of the composite material and the macroscopic damage tensor model. The new coupled damage model was proven to be reasonable in reflecting the deformation and strength of the grouted rock mass by experiment.
(2) The macroscopic damage tensor model contains the main geometric characteristic parameters of the fracture and mechanical parameters, which can be used to comprehensively describe the macroscopic damage of the fractured rock mass. On this basis, the parameter of fracture width is introduced to describe the evolution law of macroscopic damage with fracture width, thereby improving the accuracy and applicability of the damage constitutive model for fractured rock masses.
(3) The Weibull model can effectively describe the elastic stage and yield stage of the rock-deformation process. However, due to the limitations of the Weibull model itself, it is unable to accurately describe the compaction stage of the rock mass. Through the analysis of the uniaxial compression test results, the effective bearing area was introduced to optimize the Weibull model. The optimized Weibull model can accurately describe the law that holds that pores decrease and then increase with strain, which is consistent with the process of transition from the compression stage to the elastic stage, yield stage, and damage stage during the compression process of the rock mass, which is closer to reality.
(4) The grouted rock mass is a standard composite material, which conforms to the “mixing law” of the elastic modulus. The original mixing model was improved by combining macroscopic damage tensor theory with the Reuss model, and the equivalent elastic modulus of the grouted rock mass was derived.

## Figures and Tables

**Table 1:** Equivalent elastic modulus of fractured rock [53].

	Fracture Width			
	0 mm	2 mm	3 mm	5 mm
Gritstone	20.343 GPa	9.36 GPa	5.262 GPa	2.847 GPa
Mudstone	9.721 GPa	2.927 GPa	1.98 GPa	1.064 GPa
Limestone	68.882 GPa	44.992 GPa	36.888 GPa	29.661 GPa

**Table 2:** Specimens’ compressive strength and peak strain.

Compressive Strength				
Intact sample	15°	30°	45°	60°
38.9 MPa	27.1 MPa	22.76 MPa	19.57 MPa	17.26 MPa
Peak Strain				
Intact sample	15°	30°	45°	60°
1.52%	1.23%	1.22%	1.21%	1.21%

**Table 3:** Parameters for calculation.

Elastic Modulus of Rock ($E_{0}$)	m	*ε*_0_	p
2910 MPa	18	1.4%	−0.5
$\mathrm{Elastic}\;\mathrm{modulus}\;\mathrm{of}\;\mathrm{grouting}\;\mathrm{material}\;(E_{a}$)	c	d	
1880 MPa	−3.055	0.937	

## Data Availability

The data presented in this study are available on request from the corresponding author.
